# Effects of nanoparticle zinc oxide on spatial cognition and synaptic plasticity in mice with depressive-like behaviors

**DOI:** 10.1186/1423-0127-19-14

**Published:** 2012-02-03

**Authors:** Yongling Xie, Yiyi Wang, Tao Zhang, Guogang Ren, Zhuo Yang

**Affiliations:** 1 School of Medicine, Nankai University, Tianjin 300071, China; 2 Tianjin Xiqing Hospital, Tianjin 300380, China; 3 College of Life Science, Nankai University, Tianjin 300071, China; 4 Science and Technology Research Institute, University of Hertfordshire, Hatfield, Herts AL10 9AB, UK

**Keywords:** Morris water maze, long-term potentiation, depression, hippocampus, nanoparticles zinc oxide (nanoZnO)

## Abstract

**Background:**

Nanomaterials, as a new kind of materials, have been greatly applied in different fields due to their special properties. With the industrialization of nanostructured materials and increasing public exposure, the biosafety and potential influences on central nervous system (CNS) have received more attention. Nanosized zinc oxide (nanoZnO) was suggested to up-regulate neuronal excitability and to induce glutamate release *in vitro*. Therefore, we hypothesized nanoparticles of nanoZnO may lead to changes in balance of neurotransmitter or neuronal excitability of CNS. This study was to investigate if there were effects of nanoZnO on animal model of depression.

**Methods:**

Male Swiss mice were given lipopolysaccharides (LPS, 100 μg/kg, 100 μg/ml, every other day, 8 times, i.p.) from weaning to induce depressive-like behaviors. NanoZnO (5.6 mg/kg, 5.6 mg/ml, every other day, 8 times, i.p.) was given as the interaction. The mouse model was characterized using the methods of open field test, tail suspension test and forced swim test. Furthermore, the spatial memory was evaluated using Morris water maze (MWM) and the synaptic plasticity was assessed by measuring the long-term potentiation (LTP) in the perforant pathway (PP) to dentate gyrus (DG) *in vivo*.

**Results:**

Results indicated that model mice showed disrupted spatial memory and LTP after LPS injections and the behavioral and electrophysiological improvements after nanoZnO treatment.

**Conclusion:**

Data suggested that nanoZnO may play some roles in CNS of mental disorders, which could provide some useful direction on the new drug exploring and clinical researches.

## 1 Background

Nanotechnologies, which have developed and popularized at an increasing rate, exploit materials and devices with functionally organization engineered at the nanometer scale. Nanostructured materials, primarily defined by unique properties and determined interaction with other disciplines, can interact with biological systems at basic molecular levels with high specificity. Making use of this molecular feature, nanodevices can stimulate and interact with objective cells in certain ways to induce and maximize desired physiological responses [1,2]. Presently, nanoscaled materials have been widely applied in many fields such as medicine, biotechnology, energy and environmental technology [1,3-5]. Nanotoxicology and the potential effects on human body have grown in significance over recent years fueled by the surge of nanomaterial-based consumer products or building materials in the market. Since they can pass through biological membranes, nanoparticles have potential toxic effects and significant pathological consequences in human [3,6-9]. Meanwhile, they've been recognized for their potential biological utility including biological science and nanomedicine [10,11]. With the industrialization and increasing public exposure, nanosized materials have received more concern over their biosafety and underlying effects on central nervous system (CNS).

ZnO nanoparticles are amongst the most commonly utilized nanomaterials in consumer products notably on account of their unique physicochemical properties [12,13]. Some studies have indicated that nanoZnO affected functions of different cells or tissues [14,15], biocompatibility [16] and neural tissue engineering [1,2], but little was known about the influence on CNS and CNS related diseases. NanoZnO was suggested to modulate synaptic transmission *in vitro *[4] and to change the spatial cognition capability via enhancing long-term potentiation (LTP) in rats [17]. It's also suggested that exposure to nanoZnO led to a genotoxic potential mediated by lipid peroxidation and oxidative stress [18]. However, nanoZnO has been regarded as a potential utility in the treatment of cancer and/or autoimmunity due to its targeting potential [19]. This presented the potential application in treatment of diseases or some possible neuroprotection. In addition, our previous studies indicated that exposure to nanoZnO resulted in increases in current amplitude and excitability of acutely isolated rat hippocampal CA3 pyramidal neurons *in vitro *[4].

Depression, as a prevalent affective disorder, affects so many individuals in the course of their lifetimes and has overwhelming emotional and physical symptoms [20]. A study performed on behalf of the World Health Organization (WHO) has reported more than half individuals with depression around the world are not being treated, and depression would rank the second prevalent cause in term of illness induced disability by 2020 [21]. However, there is little information about the pathogenesis of depressive disorders. Disturbances of neurotransmitters, ligands and receptors and decoupling of the feedback of hypothalamic-pituitary-adrenocortical (HPA) systems with certain impairment of the immune system are supposed to be experienced during depression with anhedonia and orientation alterations towards conscience and order [22]. It's suggested that inflammation can induce abnormities in CNS, such as release of proinflammatory cytokines, excitatory amino acids and nitroxidative species which may be responsible for cognition deficits and other pathological diseases [23]. Activation of the peripheral innate immune system through the administration of lipopolysaccharide (LPS) induces depressive-like behavior that can be attenuated by chronic antidepressant administration. Both clinical reports and research data revealed that activation of the tryptophan catabolizing enzyme indoleamine 2, 3-dioxygenase (IDO) was greatly involved. Through regulating the ratio of plasma kynurenine to tryptophan, the IDO further alters serotoninergic and glutamatergic neurotransmission. Alternatively, kynurenine, as the major product of peripheral tryptophan degradation, can be transported into the brain through blood-brain barrier (BBB) and metabolized to produce neuroactive glutamatergic compounds, further resulting in the disorder of transmission system [23]. Accompanied by cognitive dysfunction, more studies are needed aiming at insights into the neuropathology of depression and offering promising alternatives or potential therapeutic action [24]. In this study, LPS repeated administrations strongly resemble the core features tested by behavioral assays, suggesting further examinations of the behavioral and neurophysiologic changes may shed light on neurobiology and treatments of depression.

According to the knowledge above, nanoZnO may play some potential role in CNS and perhaps during development processes of diseases through mediating neuronal excitability or even release of neurotransmitters. The present study was determined to see if there were effects of nanoZnO on cognitive deficits and long-term potentiation (LTP) changes in mice with depressive-like behaviors.

## 2 Methods

### 2.1 Reagents

Lipopolysaccharide (LPS, L-3129, serotype 0127:B8) was purchased from Sigma (St. Louis, MO). Before the injection, the fresh solution was prepared by diluting stock solution to final dose with sterile endotoxin-free isotonic saline and was administered intraperitoneally (i.p.). The stock solution was stored in 4°C.

The particles of nanoZnO in our research were provided by IntriciQ Materials, Farnborough, Hants GU14 0LX, UK, occurring as white powder which is nearly insoluble in water but in acids and bases. It was showed by the transmission electron microscopy (TEM, Tecnai G2 20 S-TWIN, FEI, USA) images that nanoZnO particles were with the sizes of 20-80 nm [4]. The stock solution of nanoZnO was prepared with sterile isotonic saline. The final concentration suspension was diluted and dispersed by ultrasonic vibration for 20 min before every use.

### 2.2 Animals

All animal care and use were conducted in accordance with approved institutional animal care procedures. Male Swiss mice aged 21 days old (weaning) were purchased from the Laboratory Animal Center, Academy of Military Medical Science of People's Liberation Army, and were reared in standard colony rooms in animal house of Medical School, Nankai University. Animals had free access to food and water in a controlled room with constant temperature (24 ± 2°C) and humidity (50-60%) with a normal light/dark cycle during all phases of experiments. All surgical procedures and behavioral tests took place during the light phase. Twenty-nine mice were randomly divided into four groups, which were control group (n = 7, sterile saline, 30 μg/kg, 0.9 g/100 ml, every other day), LPS group (n = 8, LPS, 100 μg/kg, 100 μg/ml, every other day), LPS+ nanoZnO group (n = 8, LPS, 100 μg/kg, 100 μg/ml, every other day, nanoZnO, 5.6 mg/kg, 5.6 mg/ml, every other day) and nanoZnO group (n = 8, nanoZnO, 5.6 mg/kg, 5.6 mg/ml, every other day). Mice were intraperitoneally injected (i.p.) respectively from weaning every other day. Injections were totally 8 times in each group. In the present study, we employed i.p. as the delivery pathway to avoid surgical damages. In addition, it's been clarified that nanoparticles through i.p. showed a biodistribution in the brain [17,25-28].

All experiments were carried out following the guidelines of the Beijing Laboratory Animal Center, and approved by the Ethical Commission at Nankai University.

### 2.3 Behavioral experiments

All behavioral experiments were performed on the day after injections. And all tests were performed in the dark room of the light cycle.

Forced swim test - the forced swim test (FST) was conducted basically and essentially as described previously [21,29]. The forced swim apparatus consisted of a cylinder container (23 cm in diameter and 31 cm in height) filled with water (maintained at 23 ± 1°C) to a depth of 15 cm. For each trial, mice were placed into the water gently and individually for 6 min and then were returned to their home cage. The water was changed between tests. During the test, the duration of immobility defined as an absence of movement necessary to keep the head above the water or climb upward-directed against the walls was determined over the last five minutes. Longer duration of immobility indicated the stronger depressive-like behavior.

Tail suspension test - we constructed the procedure of tail suspension test (TST) as described by Steru et al essentially [21,30,31]. Mice were taken from their home cage and a small piece of paper adhesive tape was attached approximately 2 cm from tip of the tail. A single hole was punched in the tape and mice were hung individually for a period of 10 min on a fixed hook. Keep the mice's heads about 20 cm above the floor. Animals would wriggle to avoid this aversive situation resulting from the suspension and body position. The time of immobility was determined at activity levels that excluded all movements and only encompassed immobility. More time of immobility indicated the stronger depressive-like behavior.

Open field test - the open field test (OFT) was referenced from procedures of Grønli et al to assess the effects on locomotor activity. The apparatus consisted of a square arena (40 cm long × 45 cm wide × 45 cm high) with a certain inner area. The floor was divided into 16 equal squares by black-colored grids. In brief, each mouse was placed in one corner of the outer area and allowed to explore for 5 min. Locomotor activity was recorded and measured by the number of line crossings and rearings over a five-min period. Counting was done by a well-trained observer who was blind to the treatments. The floor was wiped thoroughly after each trial. Fewer times of crossing and rearings indicated the stronger anxiety behavior.

### 2.4 Spatial working memory

All these procedures were performed right following all behavioral tests.

Mice were trained to learn the position of the invisible hidden escape platform using a standardized Morris water maze (MWM) procedure to monitor and evaluate their hippocampus dependent spatial learning and memory.

The equipment of MWM consisted of a tank that was 120 cm in diameter and 60 cm in height, which was filled with water to the depth of 45 cm deep and maintained at 23 ± 1°C by an automatic heater. Black nontoxic ink was added to make the water opaque. The tank was divided into four equal quadrants (I, II, III, and IV) by two imaginary perpendicular lines crossing in the center of the tank. A movable black circular platform (5 cm in diameter) was located in the center of quadrant III which is supposed to be the target quadrant. The platform submerged 1-2 cm below the water surface so that mouse could easily climb onto to escape from water. A camera located above the center of the maze and a computerized animal tracking system (Ethovision 2.0, Noldus, Wagenigen, Netherlands) were used to monitor and relay images. Data from the trials were collected for off-line analyzing. The environment was kept lightless and noiseless, maintaining visual extra-maze clue and furnishment immobile and minimizing the noise disturbance.

The MWM consisted of two sections: place navigation and spatial probe. The animals were subjected to training trials for five consecutive days. Mice were randomly placed into water facing the pool wall individually from the preset starting points. The starting location varied among four equidistant points around the perimeter of the apparatus. Subsequent starting points proceeded in a clockwise manner for the ensuing trials. Briefly, the location of the platform remained constant and mice were allowed to swim for 60 s or until they located the platform. Mice that failed to locate the platform within 60 s were guided manually to the platform and remained for at least 5 s before returning to their home cage. There was a 5 min interval among trials.

During the spatial probe, the platform was removed from the tank. Mice were released into water and allowed to swim for 60 s individually from the starting point which was in the opposite of the target quadrant. The time that each subject spent in which the platform had been located during the training was recorded.

Performance parameters in MWM determined included latency to the platform, quadrant dwell time, times of crossing and swimming speed.

### 2.5 Electrophysiological recordings

After completion of all behavioral assays, the electrophysiological recording was measured *in vivo*. Synaptic responses were quantified as the field excitatory postsynaptic potential (fEPSP) extracellularly recorded in the perforant pathway (PP) to dentate gyrus (DG) region. After anesthetized by 5% urethane (0.3 ml/10 g, i.p.), the mouse was placed in a stereotaxic frame (Narishige, Japan). At the electrode inputting region of the left side, the scalp was incised and a small hole was drilled in the skull using a dental drill. A concentric bipolar stimulating electrode was slowly implanted into the PP (2.0 mm posterior to the bregma, 1.5 mm lateral to midline, 1.5-2.0 mm ventral below the dura). A monopolar extracellular stainless steel recording electrode was positioned into DG region (0.5 mm anterior to the bregma, 3.2 mm lateral to midline, 1.5-2.0 mm ventral below the dura). During recording, the optimal depths of electrodes were determined using the electrophysiological criteria.

A stable normalized baseline was recorded for 15 min after positioning the electrodes. Then a high frequency stimulus (HFS, 10 pulses at 100 Hz for 2 s repeated 10 times) was delivered at test intensity for induction of LTP in the DG. The fEPSPs were recorded at 20 kHz sampling rate (Scope software, PowerLab, AD Instruments, Australia) every 30 s for 60 min. The fEPSPs were amplified (× 100), filtered at 5-5 kHz, digitized and recorded using Scope software (Power Lab, AD Instruments, Australia). The initial analysis of data was done in Clampfit 9.0 (Molecular Devices, Sunnyvale, CA, USA). The fEPSPs slope was measured as the average slope from 20% to 80% of the decreasing phase to measure the synaptic efficacy.

### 2.6 Statistical analysis

All data were presented as mean ± SEM and analyzed using SPSS 16.0 and Origin 8.0. Escape latencies and swimming speeds were compared using repeated measures ANOVA. Data from spatial probe and LTP recording were normalized and compared using a one-way ANOVA, followed by a *post hoc *pairwise multiple comparison procedure using the Fisher's LSD method if the interaction was significant. The behavioral results were analyzed with a two-way ANOVA. The probability level interpreted as statistically significant was *P *< 0.05.

## 3 Results

### 3.1 Changes in behavioral tests

To check depressive-like behaviors, the behavioral assays, including open field test, forced swim test and tail suspension test which have been used widely to evaluate this animal model in related researches were conducted.

Numbers of rearings and crossings in OFT were counted and compared between groups. The data were thought to reflect the abilities of exploration and spontaneous locomotion. Mice usually struggle to escape from these situations, interspersed with periods of immobility that has been interpreted as "behavioral despair" [20,32]. So the time of immobility in FST and in TST was calculated to glint changes linked to the depression directly and clinically.

**Figure 1:**
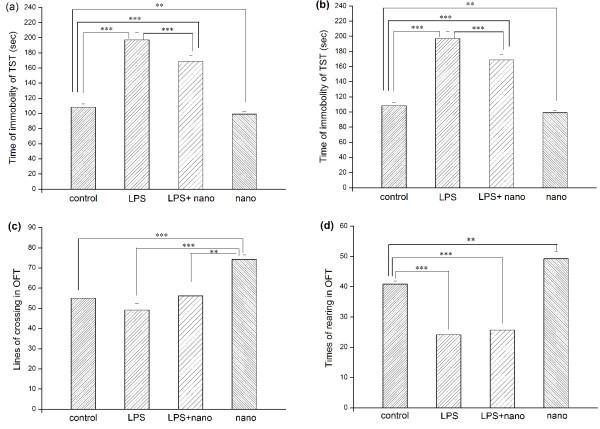
**Performances in behavioral assays**. Behavioral tests including forced swim test (FST), tail suspension test (TST) and open field test (OFT). Time of immobility was evaluated in the FST and TST as showed in A and B separately. Numbers of crossings and rearings were counted in the OFT as showed in C and D. Each data represents mean ± SEM. *P < 0.05, ** *P *< 0.01, ***P < 0.001.

Comparisons revealed that mice of LPS group took more time to keep immobile in FST than that of control (122.88 ± 6.10, 83.38 ± 2.82, *P *< 0.001) and LPS+nanoZnO group (122.88 ± 6.10, 84.16 ± 3.52, *P *< 0.001) (as shown in Figure 1.A). Mice of nanoZnO group took slightly less time to keep immobile than that of control group (69.88 ± 3.36, 83.38 ± 2.82, *P *> 0.05) at the same time, but there was no difference between LPS+nanoZnO group and control one (*P *> 0.05). In TST, there were similar results that mice of LPS group exhibited increased immobility more than that of control and LPS+nanoZnO groups with significant differences (197.25 ± 9.50, 108.38 ± 3.04, *P *< 0.001; 197.25 ± 9.50, 159.16 ± 5.13, *P *< 0.001) (as shown in Figure 1.B). In nanoZnO group, mice showed less time than that of control group (94.38 ± 3.00, 108.38 ± 3.04, *P *< 0.01).

In OFT, there were significant differences between LPS group and control group in rearings (22.00 ± 0.97, 40.88 ± 1.58, *P *< 0.001) but not in crossings (49.16 ± 2.56, 55.00 ± 1.88, *P *> 0.05) respectively (as shown in Figure 1.C and 1D). In LPS+nanoZnO group, there was significant increase in numbers of crossings than that of LPS group but not the control group (57.14 ± 2.31, 49.125 ± 2.56, *P *< 0.05; 57.14 ± 2.31, 55 ± 1.88, *P *> 0.05). Meanwhile, for the number of rearings, it displayed some different results. LPS group showed markedly reduced number of rearings than that of control group (22 ± 0.98, 40.875 ± 1.58, *P *< 0.001) but there was no significant difference between LPS group and LPS+nanoZnO group (22 ± 0.98, 25,667 ± 1.667, *P *> 0.05). Also, mice of nanoZnO group displayed significantly increased number of rearings than that of control group (49.25 ± 2.28, 40.875 ± 1.58, *P *< 0.01). While rearing numbers were significantly attenuated in LPS+nanoZnO group than those of control group. Collectively, these data strongly suggested that activation of the peripheral innate immune system with LPS administrations induced a depressive-like syndrome in mice and meanwhile the treatment of nanoZnO resulted in some improvements in behavioral response and emotional normalization to inescapable stress which might be sighted.

### 3.2 Changes of spatial cognition in MWM

To determine whether the administration of LPS efficiently induced depressive-like behaviors in mice with learning and memory deficits and if the treatment of nanoZnO particles could reverse or improve these impairments, the suspension of nanoZnO was injected in mice intraperitoneally, and changes were analyzed during the test of MWM.

**Figure 2:**
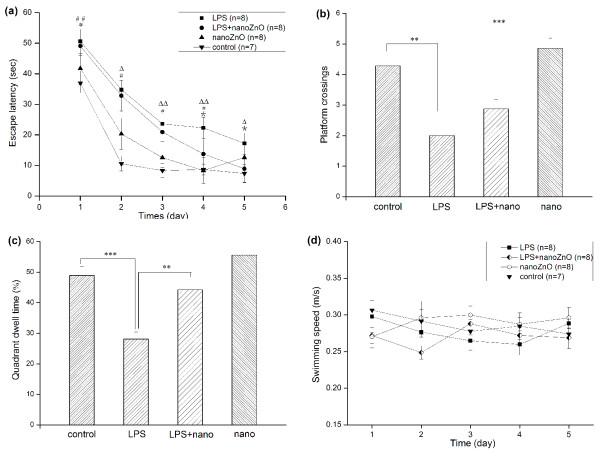
**Performances of mice in MWM**. Escape latency (A) from the place navigation session, percent of platform quadrant dwell time (B) and numbers of platform crossings (C) in spatial probe stages and the swimming speed calculated for each training session (D) were presented as mean ± SEM. There are significant differences when *P *< 0.05. In Fig.2 (A), *P < 0.05, comparison between LPS+nanoZnO vs. Control; ^## ^*P *< 0.01, comparison between LPS vs. Control; ^Δ ^*P *< 0.05, ^ΔΔ ^*P *< 0.01, comparison between nanoZnO vs. Control; * *P *< 0.05, comparison between LPS vs. LPS+nanoZnO.

In place navigation, mice of all groups took decreasing time over the course of the training to locate the submerged platform indicating that mice could definitely get and remember the location of the hidden platform through training and exhibit increasingly efficient exploratory strategies during the following trials (as shown in Figure 2.A). Overall repeated ANOVA analyses showed there were significant differences from day 1 to day 4 between LPS groups and control group (*P *< 0.01 on day 1 and *P *< 0.05 on day 2, 3 and 4). And on day 4 and 5, there were also significant differences between LPS group and LPS+nanoZnO group (*P *< 0.05). Data also showed that there were no statistical differences since day 2 until the end of the test (*P *< 0.05 on day 1).

In spatial probe trial, performances were assessed by the percentage of time spent in the maze quadrant (quadrant dwell time) and mean platform crossings (as shown in Figure 2.B and Figure 2.C). The ANOVA analyses showed that mice of LPS group failed to locate the platform in the target quadrant. Percentages of time in the target quadrant and numbers of platform crossings were less in LPS group than those of control group (28.121 ± 2.24, 48.9 ± 3.002, *P *< 0.001; 2 ± 0.408, 4.285 ± 0.333, *P *< 0.01), however there were prominent improvements in LPS+nanoZnO group especially quadrant dwell time (44.221 ± 3.24, 28.121 ± 2.24, *P *< 0.01) but not in numbers of platform crossings (2.875 ± 0.307, 2 ± 0.408, *P *> 0.05). Meanwhile, there was no significant difference in the target quadrant and numbers of platform crossings between nanoZnO group and control group (55.557 ± 3.939, 48.9 ± 3.002, *P *> 0.05; 4.857 ± 0.333, 4.285 ± 0.333, *P *> 0.05).

In addition, the swimming speeds of each group remained constant throughout the test with no significant differences between groups (*P *> 0.05; as shown in Figure 2.D). And the motor ability of animals was not changed and did not result the difference between groups. The results suggested that the treatment of nanoZnO partially reversed the learning and memory impairment.

### 3.3 Changes of LTP recording within the PP-DG pathway

Stimulation of PP evoked a basal fEPSPs in DG area and HFS induced potentiation of synaptic transmission for at least one hour in LTP recording *in vivo*.

**Figure 3:**
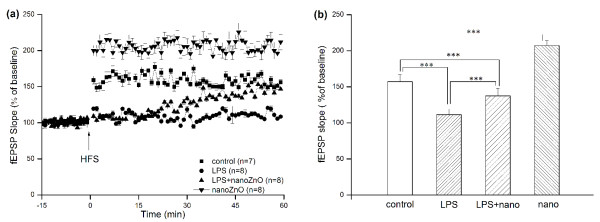
**Representative fEPSPs evoked by HFS in PP-DG area *in vivo***. fEPSPs were continuously recorded and normalized prior to HFS as baseline and 1 h after HFS as the LTP (A). Values are expressed as percentage of change relative to baseline. Magnitude of LTP, determined as responses between 30 and 60 min after HFS (B) were represented as the mean ± SEM. There are significant differences in different groups (*P *< 0.05).

To further study the mechanisms of nanoZnO particles, slopes of fEPSPs were normalized and recorded for 15 min as baseline as delineated, and after HFS, slopes of fEPSPs increased immediately and stabilized above the baseline level. The ANOVA analysis revealed that after HFS, LTP was not efficiently induced in mice of LPS group consistent with the depressed slopes (as shown in Figure 3.A). Slopes of LTP during the time course of the last 30 mins were also calculated and compared for each (as shown in Figure 3.B). It was found that the fEPSPs slopes were significantly decreased in LPS group compared with control group (110.16 ± 5.63, 157.09 ± 6.70, *P *< 0.001) and LPS+nanoZnO group (110.16 ± 5.63, 139.78 ± 8.02, *P *< 0.001), but there was a significant enhancement between nanoZnO group and control group (206.225 ± 6.06, 157.09 ± 6.70, *P *< 0.001).

## 4 Discussion

It's reported that nanoengineered materials might be used as approaches to interact with neurons to stimulate adhesion and growth and be chemically functionalized by attaching large varieties of biological molecules to cellular outer surface (for example, antibodies, peptides and trophic factors). Different nanosized particles or materials have been applied as nanoenabled drug-delivery system. By passing the BBB and translocating to the blood stream, complex can facilitate certain drugs efficiency and the release of biochemical, providing novel approaches for transporting therapeutics and targeted therapy in the treatment of brain cancer, neurodegenerative diseases, and used as a sought-after utility in nanomedicine and neuroscience [1,2,33-36]. In a different approach, nonporous silicon coated in electrodes was observed in PC12 cells to derive precursor cells, increase neurite outgrowth and decrease glial responses. Substantia nigra neurons cultured on silicon dioxide (SiO_2_) surfaces containing different nanoscale topographies had differential cell adhesion properties [1]. It's concluded that nanomaterials could influence central nervous system *in vitro *in a positive way which indicated that nanosized mental materials might act to be neuroprotective or promotive and the applications were probably correlated with physical sizes.

It's been understood that nanostructured zinc oxide have been widely applied in many aspects due to special properties and certain levels of biocompatibility. Some typical distinctive properties of nanoZnO were the capability of antibacterial [37,38], generation of reactive oxygen species (ROS) [39,40] and membrane damage possibly caused by generation of ROS [37,38,40]. They are consistent with the toxic effects that have recently received lots of concern. But little was known about its potential beneficial role either in correlational studies or in diseases. Previous research in our lab supported that nanoZnO had effects on acutely isolated rat hippocampal CA3 pyramidal neurons. By enhancing the current amplitude of *I*_Na _and *I*_K _and shifting neuronal excitability, probably mediating neurotransmitter release, nanoZnO may regulate ionic homeostasis and the physiological functions of neurons and have potential influence in CNS which shed light on the possible application and treatment in neurotransmitter system disorders in CNS [4]. Meanwhile, understanding the pathophysiological mechanisms by which inflammation and depression are linked has wide ranging implications that spans both disciplines and clinical specialties. As IDO activation was proposed to mediate a change in serotoninergic neurotransmission during the development of depression, the hypothesis was raised that nanoZnO may provide some positive benefit to the cognitive impairment or other injuries accompanied by the depressive-like behaviors. In this study data showed that nanoZnO improved the behavioral and cognitive deficits in a mice model of depression, examining by behavioral assays and electrophysiology respectively.

Open field test, tail suspension test and forced swim test are usually adopted to detect and evaluate depressive-like behaviors [21,41]. Results obtained in this study showed that mice of LPS group performed with increased durations of immobility in TST and FST and less numbers of crossings and rearings in OFT. This was associated with the decreased exploratory behaviors and locomotor abilities during depression when animals happened to be susceptible to hopeless and despair. In concert with previous reports, abilities to explore the surrounding environment and the spontaneous ability were severely impaired in LPS group representing the behavioral and emotional changes which could imitate and agree with clinical symptoms of depression. Additionally, mice of LPS+nanoZnO group appeared positive changes signing benefits resulted from administrations of nanoZnO.

Morris water maze (MWM) is a well-validated method for evaluating learning and memory. It can reliably express hippocampus-related acquisition and the persistence of spatial memory [42,43]. Results in this study revealed that with the increase of training days, escape latencies were consistently decreased and there was prominent improvement during the last two days between mice of LPS+nanoZnO group and LPS group. Meanwhile there was significant difference between LPS group and control, indicating the disruption of learning and memory during depression. This is in accordance with former reports suggesting that acquisition and retention capacities were impaired in depression model. Mice in control group learned quickly and maintained the time of locating the submerged platform after the first day of training. The learning ability was mainly reflected by the performances in place navigation section. Our data displayed that escape latency was reduced with the increase of training between groups, whereas the swimming speed remained constant suggested that motor ability was not involved in prolonged latencies. Results also suggested that treatment of nanoZnO attenuated the impairment of learning acquisition effectively in LPS group and LPS+nanoZnO group. Furthermore, the persistence of spatial memory was mainly reflected through mouse performances in spatial probe stage. It's observed that numbers of platform crossing and quadrant dwell time were markedly decreased in LPS group. However, the treatment with nanoZnO ameliorated these defects efficiently. Some previous reports suggested that chronic neuroinflammation induced direct functional impairments of synaptic plasticity underlying learning and memory process [24,44], and over-expressions of cytokines causing direct neuron injuries [45,46], which were consistent with the performance in MWM in our study. In brief, our results indicated that inflammation was sufficient to induce learning and memory dysfunction and resemble certain deficits and clinical symptoms, and administrations of nanoZnO could improve the memory ability in mice with depressive-like behaviors.

Hippocampus, conducting a major function of cognition and memory, depends on synaptic transmission properties mainly referring to the neuron network and synaptic functions [44]. By different means of identifying and characterizing, LTP usually acts as one functional index of synaptic plasticity and is one major form of experience-dependent alteration of excitatory synaptic transmission. It's definitely supposed to be the cellular mechanism underlying memory formation to manifest the strength of synaptic junctions connecting neurons and changing with experience [47,48]. Growing evidence indicates increase in the size of fEPSPs can be observed after induction by HFS, which is consistent with enhancement of synaptic plasticity and cognition [49]. Along with function of dentate gyrus (DG) in hippocampus to be understood and its important role in memory and formation of LTP focused [50-52], we recorded the LTP in PP-DG area in vivo as a major neurophysiologic index. Results showed that the normalized slope of the fEPSPs was seriously abolished in LPS group. And data obtained from our study were in agreement with the above result in MWM. Furthermore, it exhibited apparent reinforcement in slopes of fEPSPs in mice of LPS+nanoZnO group, while in nanoZnO group LTP showed significant enhancement. According to previous studies, LTP deficits in LPS group could be connected with many reasons, such as cytokines [53,54], reactive nitroxidative species (RNS) [23], or ion channel dysfunction[55]. It was suggested that cytokines or ROS/RNS could induce direct neuron injures, decrease the volume of hippocampus [56] or disturb transmitter system. Results also matched with previous outcome that nanoZnO enhanced neuron excitability [4,57] and mediated neurotransmitter release, and even regulated synaptic plasticity. Research in emerging area of nanotechnology demonstrated that the feasibility of functionalizing glass attached with inhibitory transmitter GABA (γ-amino butyric acid) through particular molecular interactions *in vitro*. Applications of nanotechnologies for neuroprotection have focused on limiting the damaging effects of free radicals generated after injury, which played an important role in neuropathological process contributing to CNS ischemia and degenerative disorders. Data (not shown) from HELC also indicated that nanoZnO treatment significantly enhanced the release of hippocampal transmitter by increasing the concentration of γ-GABA in LPS+nanoZnO group [58]. Additionally, increased prevalence of comorbid depressive symptoms appears during kinds of conditions (such as aging, obesity) and diseases (such as atherosclerosis, rheumatoid arthritis), all of which have common chronic neuroinflammation [21,59,60], undergoing over-expressions of inflammatory cytokine during the peripheral system. Cytokines modulate release of transmitters, Ca^2+ ^signal pathway and synaptic function which played an important role in physiological response and memory processing besides inflammation and nervous homeostasis [61]. Recent reports from our lab revealed that through shifting LTP to a higher level than that in the LPS group, spatial memory and cognition might be improved to some degree. What's more, the over-enhanced LTP in nanoZnO group damaged spatial cognition and capability. This bidirectional effect of nanoZnO might be explained by disrupting the balance of stability and flexibility of synaptic plasticity [17]. Certainly, the effect of nanoZnO on fEPSPs may possibly through a mechanism that is unrelated to the up-regulation of Na and K channels and still need further investigation to interpret. Briefly, our results suggested nanoZnO may benefit mice behaviorally and acts to be neuroprotective in some ways. In the light of other research, zinc was presumed to act as antidepressive and shed light on some other possible mechanisms involved in effects of nanoZnO [62,63].

Interestingly, our data are not strictly consistent prior reports. Some mentioned that nanoZnO may cause adverse effects partly due to the generation of reactive oxygen species (ROS) [37,40,64], release of zinc ions [38,65,66], membrane damage through direct nanoparticle-cell membrane wall interaction or generation of ROS [37,38,40]. With regard to nervous tissue, data showed that Neuro-2A cells and human neural cells and fibroblasts underwent apoptosis and necrosis when exposed to nanoZnO [67,68]. A recent study led by Nanyang Technological University (NTU) has found that nanoZnO could potentially cause cancer by damaging the DNA which in turn activates p53, which acts as one of the most important tumor suppressors and protects cells from developing cancer phenotypes [69].

Collectively, the dosage and particle physical diameter used in this study were proposed to be responsible for this protection and need to be investigated further. Some studies considered nanosized particles act in different ways related with particle physical sizes.

## 5 Conclusion

In summary, this study verified that zinc oxide nanoparticles could ameliorate the behavioral and cognitive impairment in mice with depressive-like behaviors, probably through up-regulating neuronal synaptic plasticity and functions in the area from perforant pathway to the dentate gyrus. More investigations will be required for a better understanding of the neuro-effect of nanosized materials and the underlying mechanisms, which provide potential therapeutic targets and clinical relevance on depression. And even more possible function mechanisms might be revealed in the further research.

## Abbreviations

CNS: central nervous system; DG: dentate gyrus; fEPSPs: field excitatory postsynaptic potentials; FST: forced swim test; FHC: bipolar stimulating electrode; GABA: γ-amino butyric acid; Glu: glutamate; HELC: High Performance Liquid Chromatography; HFS: high frequency stimulus; HPA: hypothalamic-pituitary-adrenocortical; LPS: lipopolysaccharides; LTP: long-term potentiation; MWM: Morris water maze; nanoZnO: nanosized zinc oxide; OFT: open field test; PP: perforant pathway; TST: tail suspension test; ZnO: zinc oxide

## Competing interests

The authors declare that they have no competing interests.

## Authors' contributions

YX carried out the experimental process, performed the statistical analysis and drafted the manuscript. YW participated in the experimental operation. ZY designed research with Professor TZ. GR contributed nanoparticles. All authors read and approved the final manuscript.
